# Solitude profiles and psychological adjustment in Chinese late adolescence: a person-centered research

**DOI:** 10.3389/fpsyt.2023.1173441

**Published:** 2023-07-06

**Authors:** Tong Zhou, Longyue Liao, Thuy-Vy T. Nguyen, Dan Li, Junsheng Liu

**Affiliations:** ^1^School of Psychology and Cognitive Science, East China Normal University, Shanghai, China; ^2^Department of Psychology, Durham University, Durham, United Kingdom; ^3^Department of Psychology, Shanghai Normal University, Shanghai, China; ^4^Shanghai Changning Mental Health Center, Shanghai, China

**Keywords:** solitude, late adolescent, latent profile analysis, person-centered approach, psychological maladjustment

## Abstract

**Objectives:**

From the perspective of person-centered research, the present study aimed to identify the potential profiles of solitude among late adolescents based on their solitary behavior, motivation, attitude, and time alone. In addition, to echo the paradox of solitude, we further explored the links between solitude profiles and adjustment outcomes.

**Methods:**

The participants of the study were 355 late adolescents (56.34% female, *M*_age_ = 19.71 years old) at three universities in Shanghai, China. Measures of solitary behavior, autonomous motivation for solitude, attitude toward being alone, and time spent alone were collected using adolescents' self-report assessments. The UCLA Loneliness Scale, the Beck Depression Inventory, and the Basic Psychological Needs Scales were measured as indices of adjustment.

**Results:**

Latent profile analysis revealed four distinct groups: absence of the aloneness group (21.13%), the positive motivational solitude group (29.01%), the negative motivational solitude group (38.03%), and the activity-oriented solitude group (11.83%). Differences emerged among these four groups in terms of loneliness, depressive symptoms, and basic needs satisfaction, with adolescents in the negative motivational solitude group facing the most risk of psychological maladjustment.

**Conclusion:**

Findings revealed the possible heterogeneous nature of solitude among Chinese late adolescents and provided a theoretical basis for further understanding of adolescents' solitary state.

## 1. Introduction

Solitude is defined as a state in which individuals do not interact with others, either in-person or in virtual environments (1). There has been a long-time debate about the costs and benefits of solitude, and individuals can experience solitude both positively and negatively (2). On the one hand, solitude was found to be associated with negative feelings, such as loneliness and depressive symptoms (1, 3, 4). On the other hand, it was believed that positive experiences with solitude could promote self-discovery, creativity, and self-reflection (1).

As late adolescents are far away from families (e.g., embrace the university), it would be important for them to form peer relationship and romantic relationship during the social transition (5, 6). However, late adolescence is also considered to be an important and unique developmental period for solitude (7). During this period, individuals would engage in solitude for completing the corresponding developmental tasks, such as autonomy from parents and identity formation and self-regulation (8). From early to late adolescence, it has been argued that solitude becomes more adaptive (9) and adolescents are more able to enjoy solitude (8) and have more positive attitudes toward aloneness (10). In addition, Eastern Asian societies may place special importance on solitude as it provides time and space for self-reflection, a practice that originated in Taoist and eremitic traditions (11). As such, some Chinese late adolescents may benefit from their solitude experience.

However, despite such theoretical postulation and research evidence on the benefits of solitude, having too much time alone may yield negative consequences. For instance, it has been found that a preference for solitude was positively associated with depressive symptoms among Chinese college students (*M*_age_ = 21.43 years) (12). Moreover, one study found among Chinese late adolescents (*M*_age_ = 19.89 years), preference for solitude was positively associated with mobile phone addiction, and such a relationship was mediated by psychological distress (13). Besides, as compared with the norm, adolescents who consistently withdraw from opportunities for peer interactions (age 18–29 years) reported higher levels of depressive symptoms (14). These findings argue that a solitary behavior may contravene values of interdependence and social harmony in the Chinese context (15).

Such inconsistency between the positive and negative sides of solitude reveals a conflicting picture of the potential significance of solitude during adolescence. One possible explanation for this inconsistency is that previous studies that explored solitude mostly applied a variable-centered approach, which captured only one dimension of solitude (e.g., preference for solitude) and failed to acknowledge other components of solitary experiences (16, 17). Understanding whether a person prefers spending time alone over interacting with other people gives little insight into what goes on during solitary states. In other words, different dimensions of solitude may interrelate with each other and configure within an individual's solitary experience in meaningful ways (i.e., the patterning). For example, the reasons why adolescents choose to spend time alone and their behaviors when alone may contribute to whether adolescents could enjoy being alone or not. Such possible patterns of different solitude dimensions may hold unique implications for individual development that cannot be accounted for by any single dimension of solitude. Using a person-centered approach, researchers can reveal the potential heterogeneity of solitude that might exist within the focal population (i.e., late adolescence in the current study) (18) and therefore obtain a better nuanced understanding of solitude. As such, we argued that a person-centered approach should be applied to provide further insights into whether there are distinct groups of adolescents who would perform certain activities and associate with different psychological outcomes while alone.

In line with the potential heterogeneity of solitude, previous researchers argued that solitude is a “complex and multifaceted” concept that includes emotion, cognitive, and behavioral dimensions (19–22). For instance, Larson (8) suggested that, when people spend time alone, they could experience both internal psychological processes, such as emotions and cognitions, and external activities. Long et al. (21) argued that “feelings, activities, and/or outcomes” constituted one's experience of solitude. To elaborate on such multifaceted nature of solitude, the current study would describe solitude from the perspective of motivation (23, 24), attitude (10, 16), behavior (25), and time duration (26).

Motivation for solitude has been studied from the perspective that builds on the self-determination theory (SDT) (27, 28). From this perspective, Thomas and Azmitia (28) categorize motivation for solitude into two types: not self-determined solitude and self-determined solitude. Not self-determined solitude represents reasons for being alone that are rooted in discomfort and negative feelings toward being with other people. However, self-determined solitude represents reasons for being alone that are driven by desires to connect with oneself and to seek privacy, calmness, and freedom. Similarly, building on self-determination theory, Nguyen et al. (24) also suggested that some solitude could be driven by intrinsic (i.e., generally enjoy solitude) and personally meaningful (i.e., value alone time for its benefits) reasons, while others might be driven by social pressure (i.e., feel they should be alone) and external influence (i.e., feel forced into solitude). Previous studies have found that more self-determined motivation for solitude was linked to psychological wellbeing, such as greater self-esteem and a sense of relatedness to others (24), while the less self-determined motivation for solitude was associated with ill-being, such as loneliness, depressive symptoms, and social anxiety (24, 28).

To understand why some people spend more time alone than others, other researchers suggest that people have different *attitudes toward solitude*; that is, a person either likes to spend time in solitude or tries to avoid it. From this perspective, Burger (16) conceptualized *preference for solitude* as the tendency to either prefer spending time or doing activities alone over being with other people. Marcoen and Goossen (29) expanded such an idea and proposed two distinctive attitudes toward solitude that is the affinity for aloneness and aversion to aloneness. However, the literature that relies on conceptualization of motivation for solitude based on approach-avoidance dichotomies often found that favoring solitude over social interactions is often associated with negative outcomes. From early adolescence to late adolescence, the affinity for solitude increases, and the aversion to solitude decreases (10). Compared with those who held aversion to solitude, adolescents who held affinity for solitude were less liked by their peers, more easily victimized during peer nominations, and scored lower on friendship quantity and quality (30).

In solitude, people more often engage in some types of *activities*, because people would feel more comfortable when they do something (e.g., have an activity to choose from) than nothing when alone (e.g., think) (31). Accordingly, Ruiz-Casares (32) investigated what activities adolescents (ages 10-17 years) engage in at home alone and found that the most common solitary behaviors include watching TV, surfing the Internet, doing homework, and playing games. A recent study coded and categorized adolescents' self-reported solitary behaviors, finding that there were three different subgroups of solitary behaviors among adolescents: thinking (e.g., daydreaming, 15.0%), passive media (e.g., passive screen and homework, 53.3%), and engaged (e.g., reading, homework, and music listening, 31.7%), with the thinking group reporting more loneliness and negative effect than those in engaged group (33).

Last but not least, the amount of *time spent alone* would also be considered as an important aspect of the adolescents' psychological implication of solitude. In earlier studies, Larson (3) first applied empirical methods (i.e., experience sampling) to investigate the effects of alone time on adolescent psychological adjustment. Specifically, it was found that spending an intermediate amount of time alone was correlated with adolescents' better adjustment than spending little or a great deal of time alone. In addition, this study suggested that when the time spent alone become an overall response tendency, it could evolve into a “misanthropy effect” that was detrimental to adolescents' mental health (3). In line with such idea, one study showed that the longer time spent alone per day would predict lower levels of adolescents' positive affect and satisfaction with life (34). However, it was also proposed that perceptions of not spending enough time alone could also be linked to adolescents' negative feelings (26).

The current study seeks to extend prior literature by exploring the different dimensions of solitude (i.e., motivation, attitudes, behaviors, and time duration) that may be configured within adolescents' solitary experience, and how such configurations may be linked to adolescents' psychological well-being and ill-being. To do this, we used latent profile analysis to examine variations in the extent to which adolescents experience solitude in their actual behaviors (i.e., solitary behavior), motivations (i.e., autonomous motivation for solitude), attitude (i.e., affinity for and aversion to aloneness), and time spent alone. Similar person-centered perspective has been used in previous research. Lay and colleagues (2) used multilevel latent profile analysis to identify two solitude groups [i.e., one positive (56.7%) and one negative (43.3%)] in adults' daily life. Maes et al. (30) adopted cluster analysis and identified six solitude groups on the basis of adolescents' loneliness (i.e., parent- and peer-related) and attitudes toward aloneness (i.e., positive and negative), with three groups displaying adaptive patterns and the other three showing maladaptive patterns. Specifically, the indifference group (17–23%, with rather low scores on the four constructs), the moderate group (18–25%, with moderately low scores on the four constructs), and the negative attitude toward aloneness group (16–21%) were considered to be adaptive. On the other hand, maladaptive pattern was found for adolescents in the peer-related loneliness group (12–19%), the parent-related loneliness group (9–16%), and the positive attitude toward aloneness group (10–14%) (30). These two findings revealed the possible heterogeneous nature of solitude and explain why some people could benefit from solitude, while others may feel lonely when being alone. However, these two person-centered studies only explored one certain dimension of solitude (i.e., cognitive effort thought, attitudes toward solitude) with the combination of the solitary affection, with neither of them considering other dimensions of solitude, such as motivation, behavior, and time.

Given the limitation of previous studies, the present study focused on four dimensions of solitude that have been studied in solitude literature, including motivation and attitudes toward solitude, and behaviors and time duration in solitude. We expected that at least one group would adjust well to solitude, that is, those who exhibit high autonomous motivation for solitude, high solitary behavior (i.e., engage in activities instead of doing nothing), and moderate time duration for being alone. At least one another group may suffer from solitude, exhibiting low autonomous motivation for solitude, high aversion to aloneness, low solitary behavior, and high time duration for being alone. Although these general trends were expected, the nature of latent profile analysis precluded specific hypotheses about the numbers of groups and precise descriptions of these groups.

Further, relatively little attention has been given to the implications of being in different solitude profiles. As such, the second goal of this study was to determine how these different profiles relate to adolescents' psychological adjustment (i.e., loneliness, depressive symptoms, and basic needs satisfactions). It was anticipated that adolescents who had a positive experience in solitude would have higher levels of psychological adjustment, whereas those who suffered from being alone were expected to have a lower level of psychological adjustment.

## 2. Materials and methods

### 2.1. Participants and procedures

Participants were enrolled in their freshman and sophomore years at three universities in Shanghai, China. The study procedure was approved by the Shanghai Normal University. A web platform, Wenjuanxing, was used to collect data. A total of 444 students were invited to participate in this study with an informed consent form on their psychology course. The consent rate was 79.95%, and the final sample includes 355 adolescents (155 male and 200 female, *M*_age_ = 19.71, *SD*_age_ = 1.02). Overall, 70% of adolescents came from urban areas of China, and 45% reported both of their parents having a bachelor's degree or more. Participants who completed the survey received additional course credit.

### 2.2. Measurement

#### 2.2.1. Solitary behavior

Participants were asked how often they take part in different behaviors when they are alone on a 5-point scale (from 1 = “never” to 5 = “always”). The average score of the responses was calculated, with higher scores indicating more frequency of activities when adolescents spend time alone. The items of this measure partially came from previous findings in children (25) and adolescents (33), and semi-structured interviews were conducted to expand the diversity of solitary behavior among Chinese late adolescents. The interviews were conducted with a sample of 12 Chinese late adolescents (aged between 18 and 20 years). According to the interviews, some of the previous items were combined with new items to create an adolescent solitary behavior measure. This measure was pilot tested with a sample of 228 Chinese late adolescents (84 male and 144 female, *M*_age_ =19.69, *SD*_age_ = 1.02) in similar geographic areas (i.e., universities in Shanghai, China) to the large majority of participants in the current sample. Items were revised or replaced based on theoretical considerations (21) and statistical issues in exploratory factor analysis (35), yielding the current measure. According to the previous theoretical suggestion (21), the four factors in adolescent solitary behavior measure were defined as self-reflection (seven items, e.g., “When I am alone, I would like to think about my future”), problem-solving (four items, e.g., “When I am alone, I would like to complete my homework”), physical activities (four items, e.g., “When I am alone, I would like to go to the gym”), and leisure browsing (four items, e.g., “When I am alone, I would like to browse social networks”), respectively (see items details in Appendix Table). To further confirm the construct of this measure, confirmatory factor analyses (CFA) were conducted in the current sample (*N* = 355). The CFA model yielded a good fit (χ^2^(144) = 297.36, *p* < 0.01, CFI = 0.94, RMSEA = 0.05, 90% CI [0.04, 0.06], SRMR = 0.06), with the loading ranging from 0.91 to 0.40. The internal reliabilities were 0.91, 0.87, 0.72, and 0.71 for self-reflection, problem-solving, physical activities, and leisure browsing, respectively, in the current sample.

#### 2.2.2. Motivation for solitude

We measured motivation for solitude using the 8-item revised Self-Regulation Questionnaire (36) developed by Nguyen et al. (24). Participants were asked about the reason for solitude (eight items, e.g., “I spend time alone because I value time alone as an important part of my day”). All the items used a seven-point scale, ranging from 1 (not at all true) to 7 (very true). The results of CFA indicated that the good structural validity (χ^2^(12) =30.38, *p* < 0.01, CFI = 0.98, RMSEA = 0.07, 90% CI [0.04, 0.09], SRMR = 0.02) and the internal reliability was 0.70 in the current study. As followed by previous practice (24), a Relative Autonomy Index (RAI) was calculated (24), with higher scores indicating more autonomous motivation for spending time alone.

#### 2.2.3. Attitude toward aloneness

The Loneliness and Aloneness Scale for Children and Adolescence [LACA; (37)] was used to measure adolescents' attitudes toward solitude. Two subscales captured aversion to aloneness (LACA-negative, e.g., “When I am bored, I feel lonesome”) and affinity for aloneness (LACA-positive, e.g., “Being alone makes me take up my courage again”). All the items used a 4-point scale, ranging from 1 (never) to 4 (often). The average score of the responses was calculated, with higher scores indicating more negative or more positive attitudes toward being alone. The measure has been used and proved to be reliable and valid in Chinese samples (38). The results of CFA in the current sample indicated good structural validity (χ^2^(53) = 117.39, *p* < 0.01, CFI = 0.95, RMSEA = 0.06, 90% CI [0.04, 0.07], SRMR = 0.05). Internal reliabilities were 0.89 and 0.79 for each subscale in the present study.

#### 2.2.4. Time spent alone

Two questions were used to measure the participants' time spent alone, referring to previous practice (26), “In the past week (7 days), how many times did you spend time alone by yourself for at least 15 minutes? (from “1 = not once” to “6 = more than 4 times a day” and “In the past week (7 days), approximately how long did you spend time alone? (from “1 = less than 7 h (less than 1 h per day)” to “6 = more than 35 h (more than 5 h per day).” The average score of the two questions was calculated as the time spent alone. Consistent with the previous study (26), these two items were highly correlated (*r* = 0.65, *p* < 0.001) and the internal reliability was 0.79 in the current study.

#### 2.2.5. Loneliness

The UCLA Loneliness Scale (39) was used to measure adolescents' loneliness as one of the indicators of psychological maladjustment. Participants were asked about their experience of loneliness in 2 weeks (10 items, e.g., “I feel no one to talk to.”). All the items used a 4-point scale, ranging from 1 (never) to 4 (always). The average score of the responses was calculated, with higher scores indicating greater loneliness. Previous studies have shown that the scale has good reliability and validity among Chinese late adolescents (12). The results of CFA indicated a good structural validity of the UCLA Loneliness Scale for the current sample (χ^2^(34) = 118.20, *p* < 0.01, CFI = 0.93, RMSEA = 0.08, 90% CI [0.07, 0.10], SRMR = 0.05). In addition, the internal reliability for loneliness was 0.89 in the present study.

#### 2.2.6. Depressive symptoms

The Beck Depression Inventory-II [BDI-II, (40)] was used to examine the depressive symptoms of the participants. The scale consisted of 20 items (e.g., “I feel like a total failure.”), which used a 4-point scale, with higher scores indicating higher levels of depression. The scale was widely used in the Chinese cultural context and had high reliability (41). The results of CFA indicated a good structural validity of BDI for the current sample (χ^2^(170) = 273.45, *p* < 0.01, CFI = 0.94, RMSEA = 0.04, 90% CI [0.03, 0.05], SRMR = 0.05). The internal reliability for depression in this study was 0.90.

#### 2.2.7. Basic psychological needs

The Basic Psychological Needs Scale (BPNS) (42) was used to examine whether the individuals' basic psychological needs were met and was revised into the Chinese version in a previous study (43). The scale consisted of 19 items that captured three basic psychological needs, which were competency needs (e.g., “I have recently been able to learn interesting new skills”), autonomy needs (e.g., “I am usually very happy to express my thoughts and opinions”), and related needs (e.g., “I really like the people I get along with”), respectively. All items were graded on a 7-point Likert scale, with higher scores indicating that the basic psychological needs were satisfied better. The scale had shown high reliability in the Chinese sample (44). The results of CFA indicated an acceptable structural validity of the BPNS for the current sample (χ^2^(114) = 332.61, *p* < 0.01, CFI = 0.90, RMSEA = 0.07, 90% CI [0.06, 0.08], SRMR = 0.04). The internal reliability for psychological needs in this study was 0.82.

### 2.3. Statistical analysis

**Figure 1:**
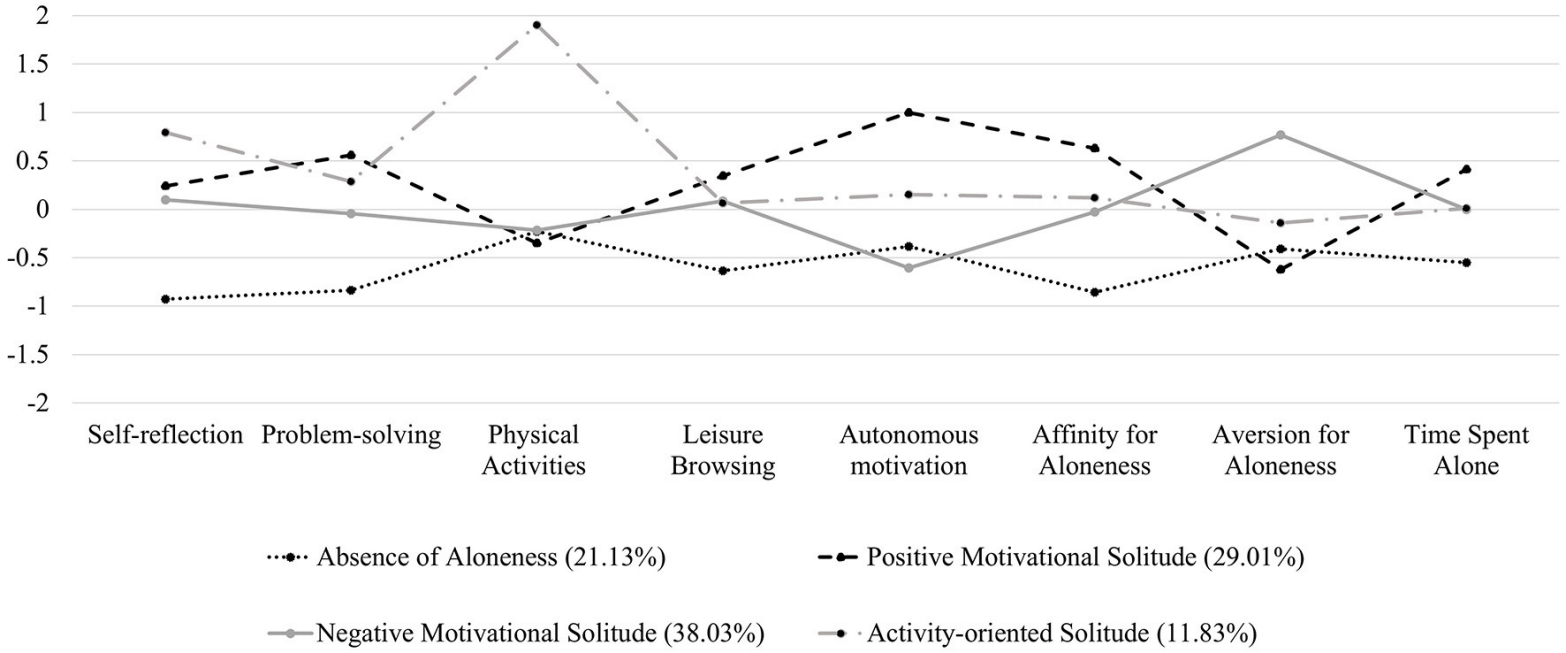
Response patterns across 8 variables for solitude profiles.

Latent profile analysis (LPA) was conducted to investigate how the observed heterogeneity in a group can be traced back to underlying homogeneous subgroups (or profiles) (45). This person-centered approach is based on the characteristics of indicators to identify different types of profiles and determines which profile an individual belongs to with a certain degree of probability (46). Compared with traditional clustering methods (e.g., k means clustering, hierarchical clustering), this probability-based mixture model outperformed in detecting potential classifications (47). In the current study, the profiles were identified based on eight variables on adolescents' solitude (see Figure 1 for more details). Specifically, we used solitary behavior (i.e., self-reflection, problem-solving, physical activities, leisure browsing), autonomous motivation for solitude (i.e., solitude RAI), attitude toward aloneness (i.e., affinity and aversion), and time alone as indices to describe adolescents' solitude experience. All eight variables were assessed using standardized units to facilitate interpretation.

As suggested by Nylund, Asparouhov, and Muthén (48), the bootstrap likelihood ratio test (BLRT), the adjusted BIC (aBIC), the adjusted Lo–Mendell–Rubin likelihood ratio test (LMRT), the Bayesian Information Criterion (BIC), and Akaike's Information Criterion (AIC) were considered as the model fit indices to determine the number of latent profiles in the present analysis. A high level of entropy indicates a greater accuracy of classification (49). A lower level of AIC, BIC, or aBIC indicates a better fitting model (48). In addition, BLRT and LMRT allow examining whether including one more latent profile significantly improves the model fit. If this is not the case, the more parsimonious model with fewer latent profiles should be selected (50). In selecting the final model, we took into consideration how well a solution could be interpreted, that is, whether the latent profiles in a solution showed logical patterns, were distinct from the other profiles, and could readily be labeled.

Finally, once the number of profiles has been determined, multivariate analysis of variance (MANOVA) was performed to see how the profiles varied from one another for each indicator. BCH analysis (51) was used in *Mplus* to examine the difference in adolescents' psychological adjustment outcomes (i.e., loneliness, depressive symptoms, basic needs satisfaction) across profiles.

## 3. Results

### 3.1. Descriptive analyses of main variables

Descriptive statistics and intercorrelations among all study variables are presented in Table 1. Moderate significant correlations were found between loneliness, depressive symptoms, and basic needs satisfaction, suggesting that these three variables could be viewed as indicators to measure adolescents' psychological maladjustment. Self-reflection was positively correlated with autonomous motivation for solitude, attitude toward solitude (affinity and aversion), loneliness, and depressive symptoms. Problem-solving was positively correlated with autonomous motivation for solitude, affinity for aloneness, time spent alone, and basic needs satisfaction. Physical activities were positively correlated with basic needs satisfactions and negatively correlated with depressive symptoms. Leisure browsing was positively correlated with autonomous motivation for solitude, an affinity for aloneness, and depressive symptoms. In addition, autonomous motivation for solitude was positively correlated with an affinity for aloneness and negatively correlated with an aversion to aloneness. Time spent alone was positively correlated with autonomous motivation for solitude and an affinity for aloneness but had no association with aversion to aloneness.

**Table 1:** Descriptive statistics and bivariate correlations for study variables (*N* = 355).

	**1**	**2**	**3**	**4**	**5**	**6**	**7**	**8**	**9**	**10**	**11**
1. Self–reflection	–										
2. Problem–solving	0.30^**^	–									
3. Physical activities	0.26^**^	0.09	–								
4. Leisure browsing	0.20^**^	0.40^**^	−0.07	–							
5. Autonomous motivation (RAI)	0.15^**^	0.27^**^	−0.02	0.14^**^	–						
6. Affinity for aloneness	0.39^**^	0.25^**^	0.02	0.15^**^	0.34^**^	–					
7. Aversion to Aloneness	0.12^**^	−0.09	0.01	0.06	−0.41^**^	0.02	–				
8. Time spent alone	0.08	0.19^**^	−0.01	0.06	0.17^**^	0.31^**^	−0.01	–			
9. Loneliness	0.24^**^	−0.03	−0.04	0.04	−0.30^**^	0.24^**^	0.58^**^	0.18^**^	–		
10. Depressive symptoms	0.23^**^	−0.06	−0.15^**^	0.18^**^	−0.13^**^	0.22^**^	0.29^**^	0.17^**^	0.46^**^	–	
11. Basic needs satisfaction	−0.08	0.22^**^	0.16^**^	0.01	0.25^**^	−0.20^**^	−0.32^**^	−0.21^**^	−0.55^**^	−0.46^**^	–
*M*	3.15	3.56	1.83	3.66	11.55	2.68	2.26	3.48	2.06	1.52	4.5
*SD*	0.81	0.75	0.72	0.68	10.64	0.59	0.73	1.49	0.62	0.44	0.63
Range	1–5	1–5	1–5	1–5	– 36–36	1–4	1–4	1–6	1–4	1–4	1–7

** *p* < 0.01. RAI, Relative Autonomy Index.

### 3.2. Adolescents' solitude latent profile identification

Table 2 shows the fit indices of the different profile models of LPA. The decline of AIC and aBIC plateaued after four profiles, and BIC increased after four profiles. The BLRT remained significant with the increasing profiles, and the LMR-LRT reached insignificance with five profiles, suggesting that the five-profile model did not fit better than the four-profile model. Besides, the indices of classification quality (Entropy) suggested a better separation of individuals into four profiles compared with three or five profiles. Accordingly, the four-profile solution was deemed optimal. MANOVA was conducted for eight solitary indicators to detect between-profile differences, and the result is presented in Table 3. The *post-hoc* analyses (LSD) revealed that adolescents in different profiles had significant differences in their classification variables. The latent profile parameters and the profile-conditional parameters (the standardized values of solitary activities, motivation, attitude, and time) are shown in Figure 1.

**Table 2:** Latent profile models and fit indices.

**Profile**	**Proportions based on the estimated model entropy (%)**						**Entropy**	**AIC**	**BIC**	**aBIC**	***p* LMRT**	***p* BLRT**
	**Class 1**	**Class 2**	**Class 3**	**Class 4**	**Class 5**	**Class 6**						
1	-	-	-	-	-	-		8083.56	8145.51	8094.75	-	-
2	48.45%	51.55%	-	-	-	-	0.62	7907.95	8004.75	7925.44	0.036	< 0.0001
3	21.69%	43.94%	34.37%	-	-	-	0.68	7830.37	7962.02	7854.16	0.009	< 0.0001
**4**	**21.13%**	**29.01%**	**38.03%**	**11.83%**	**-**	**-**	**0.74**	**7756.14**	**7922.64**	**7786.23**	**0.005**	**< 0.0001**
5	19.16%	15.78%	39.72%	11.55%	13.80%	-	0.73	7739.71	7941.06	7776.09	0.381	< 0.0001
6	17.47%	13.80%	42.82%	11.27%	13.52%	3.66%	0.76	7729.69	7965.89	7772.37	0.573	0.072

Values in bold type indicate the chosen model in the current study.

AIC, Akaike information criterion; BIC, Bayesian information criterion; aBIC, the adjusted Bayesian information criterion; LMR, Lo–Mendell–Rubin likelihood ratio test; BLRT, bootstrapped likelihood ratio test.

**Table 3:** Results of descriptive data for each group and *post-hoc* comparisons (MANOVA).

	**SR**	**PS**	**PA**	**LB**	**AM**	**AFA**	**ATA**	**TSA**
Absence of aloneness	2.32 (0.57)_a_	2.89 (0.65)_a_	1.68 (0.54)_b_	3.22 (0.68)_a_	7.92 (7.99)_b_	2.14 (0.43)_a_	1.89 (0.47)_a_	2.55 (1.22)_a_
Positive motivational solitude	3.34 (0.75)_b_	4.01 (0.66)_d_	1.56 (0.44)_a_	3.93 (0.66)_c_	22.41 (6.62)_d_	3.06 (0.56)_c_	1.78 (0.59)_a_	4.10 (1.46)_c_
Negative motivational solitude	3.26 (0.66)_b_	3.51 (0.63)_b_	1.70 (0.52)_b_	3.67 (0.62)_b_	4.51 (7.55)_a_	2.65 (0.47)_b_	2.86 (0.55)_c_	3.46 (1.41)_b_
Activity-oriented solitude	3.81 (0.62)_c_	3.78 (0.56)_c_	3.24 (0.50)_c_	3.71 (0.52)_bc_	14.07 (8.75)_c_	2.76 (0.50)_b_	2.12 (0.55)_b_	3.71 (1.42)_bc_

SR, Self-reflection; PS, Problem-Solving; PA, Physical Activities; LB, Leisure Browsing; AM, Autonomous Motivation; AFA, Affinity for aloneness; ATA, Aversion to aloneness; TSA, Time spent alone. Different subscripts in the same column indicate significant differences from one another (*p* < 0.05); a < b < c < d.

The four solitary latent profiles are described and named according to the salient features exhibited by the observed indicators, as follows: (1) **absence of aloneness profile** (Profile 1, 21.13%) was characterized by the lowest levels on all indicators of solitude (except aversion to solitude) compared with other three groups, showing that the participants of the profile would spend little time alone. (2) **positive motivational solitude profile** (Profile 2, 29.01%) was characterized by the highest level of autonomous motivation, an affinity for aloneness, time spent alone, and problem-solving, implying that participants of this profile would prefer solitude to intrinsic motivation and be possible to solve problems. (3) **negative motivational solitude profile** (Profile 3, 38.03%) was characterized by the highest level of aversion to aloneness and the lowest level of autonomous motivation, which displayed that participants of this profile may dislike being alone but have to stay alone for extrinsic reasons. (4) **activity-oriented solitude profile** (Profile 4, 11.83%) was characterized by the highest level of physical activities and self-reflection.

### 3.3. Differences between latent profiles in psychological adjustment

With respect to the psychological adjustment, we examined the difference between each latent profile (Table 4). The results of BCH analyses indicated significant differences between groups. Specifically, the loneliness of the *negative motivational solitude* group was significantly higher than those in the remaining three groups, whereas adolescents in the *absence of aloneness* group obtained the lowest loneliness. In addition, there were no significant differences in loneliness between adolescents in the *positive motivational solitude* and *activity-oriented solitude* groups.

**Table 4:** Comparison of psychological adjustments among different solitude groups *M* (*SD*).

	**Loneliness**	**Depressive symptoms**	**Basic needs satisfactions**
Absence of aloneness	1.70 (0.47)_a_	1.35 (0.34)_a_	4.58 (0.60)_b_
Positive motivational solitude	1.87 (0.53)_b_	1.50 (0.41)_b_	4.62 (0.69)_b_
Negative motivational solitude	2.43 (0.57)_c_	1.68 (0.49)_c_	4.28 (0.51)_a_
Activity-oriented solitude	1.95 (0.62)_b_	1.35 (0.30)_a_	4.75 (0.72)_b_

Different subscripts in the same column indicate significant differences from one another (*p* < 0.05), a < b < c.

A similar pattern has been found in the other indices of psychological maladjustment, adolescents in the *negative motivational solitude* group obtained the highest level of depressive symptoms than the other three groups, whereas adolescents in the *absence of aloneness* group and the *activity-oriented solitude* group obtained the lowest level of depressive symptoms than the other two groups. In addition, adolescents in the *positive motivational solitude* group scored higher on the level of depressive symptoms than adolescents in the *activity-oriented solitude* group.

Finally, adolescents in the *negative motivational solitude* group exhibited the lowest level of basic needs satisfaction than the rest of the three groups, and there were no differences in the level of basic needs satisfaction among the remaining groups.

## 4. Discussion

The cost and benefit of solitude have long been researched in developmental studies. Owing to the complex and multifaceted characteristics of solitude, the current study embraced a person-centered approach to offer a new perspective on solitude among late adolescents. We aimed to first identify the naturally existing solitude group in Chinese late adolescents and to determine to what extent adolescents' behavior, motivation, attitude, and time when being alone would perform within a solitude profile. In addition, the second goal was to explore the association between different profiles and adolescents' psychological adjustment. Four distinct profiles were identified via latent profile analysis, and their prevalence was documented.

First, it was found that, among the four profiles, only the *negative motivational solitude* group experienced psychological maladjustment (i.e., high levels of loneliness, depressive symptoms, and low levels of basic needs satisfaction). Adolescents in this profile showed the highest level of aversion to solitude and the lowest level of autonomous motivation for solitude, suggesting the possibility that they disliked being alone but had to be alone. In the current sample, adolescents in this profile may not accept their current state of being alone, consider solitude to be worthless, and may also not believe that they were capable of being alone. Based on self-determination theory (27), non-self-determined experiences would put individuals at risk for psychological maladjustment. Previous studies have also shown that not self-determined solitude can lead to loneliness and other psychological problems (52). It was noteworthy that this profile accounted for 38.03% of the sample, with the highest percentage among those four profiles, supporting the view that solitude was risky and should be avoided to some extent for adolescents (53).

Further, the present study found that there was more than one answer regarding the extent to which adolescents could benefit from solitude. Two profiles were both correlated with good psychological adjustment, while they had different characteristics. Adolescents in the *positive motivational solitude* profile exhibited the highest level of autonomous motivation, an affinity for aloneness, the time spent alone, and problem-solving, suggesting that they may voluntarily prefer solitude and were more likely to solve problems when being alone. This group represented around 30% of our sample. Similar results were found in another study (30), in which early adolescents exhibited a preference for solitude, accounting for 30.36% and 26.10% in the two samples. Consistent with previous findings (24), people tend to benefit from solitude for autonomous motivation and are not disturbed by intrusive negative thoughts. However, when we attempt to understand the well-adjusted psychological outcomes of these motivation-driven groups, it is also noteworthy to combine with the interpretation of attitude toward solitude in our findings. Specifically, previous studies have found that a preference for solitude was associated with loneliness and psychological maladjustment among *early* adolescents (38, 54). In the current study, the affinity for aloneness was exhibited together with autonomous motivation for solitude, which brought well psychological outcomes for *late* adolescents. Such finding suggested that, on the one hand, the hypothetical framework of developmental time effect of solitude (9) has been supported. Older adolescents are troubled less when they prefer to be alone. On the other hand, the person-centered approach helped us find the significance of combining motivation and behavior to interpret attitudes toward solitude among late adolescents.

A newly found well-adapted group was labeled as the *activity-oriented solitude* group, which was characterized as the highest level of solitary behaviors (i.e., physical activities and self-reflection). This group encompassed around 12% of the sample and had the lowest proportion of all profiles, suggesting a relatively small proportion of adolescents that may exhibit such characteristics when they spent time alone. Such a result echoed previous findings (31) to some extent, suggesting that people may enjoy doing mundane external activities more than doing nothing when they spend time alone. The positive relationship between exercise and mental health has been widely supported in previous findings (55), thus, it is not difficult to understand why late adolescents in this group are well-adjusted. Although there was no direct evidence in previous studies about exercise alone, researchers found that leisure activities, such as hiking and walking in the wild, were involved in an individual's solitary experience (56). As such, the emergence of the activity-oriented solitude group may indicate specific types of behaviors that adolescents can engage in to have a positive experience when alone.

Finally, although the current research has focused on adolescents' experiences of being alone, a profile characterized by low levels of solitude was identified. We named this group as the *absence of aloneness*, as it showed the lowest score in seven of the eight solitary indicators. This profile represented 21.13% of the sample, which was consistent with previous findings (30). Specifically, a group of adolescents were found, showing a low affinity for solitude and a low aversion to solitude, accounting for 23.08%, 24.27%, and 16.96% in three different samples. Such findings could be interpreted as there being a group of adolescents who neither like nor hate solitude and rarely choose to be alone. Their daily life may be filled with interpersonal activities in general, and therefore, they are more well adjusted than other groups. However, it should be noted that, because we did not measure the level of adolescents' social interactions in the current study, the absence of aloneness cannot be directly equated with having a more active social life. Instead, it is possible that adolescents in the absence of aloneness group may have ambivalent attitudes toward their alone time because the possibility of being alone is relatively low in their life. At least for this sample, we did not observe evidence suggesting that the lack of any intention or attitudes toward solitude yields negative wellbeing consequences for late adolescents.

## 5. Limitation and future direction

In this study, we considered solitude as a multifaceted structure and applied a person-centered approach to identify four solitary profiles. The proportion of the negative motivational solitude profiles and the possible manifestations of solitude were revealed. Besides, we also discovered two profiles that may benefit from solitude and their characteristics. The study enriches and integrates previous findings to some extent and also provides new perspectives for understanding the phenomenon of solitude among Chinese late adolescents.

However, there are several limitations in the present study. First, considering the developmental time effect of solitude (9) from early to late adolescents, future studies may consider applying a person-centered approach at different developmental stages of adolescence (e.g., early and middle) to further explore the possible heterogeneous nature of solitude and its relationship with adolescents' developmental outcomes.

In line with such an idea, a developmental perspective could also offer us more information on the paradox of solitude in future. The current study applied a cross-sectional design, and little was known about dynamic developmental changes in solitude among adolescents. From the developmental perspective, more research questions remain to be answered. For example, do those distinct solitude groups perform the same across developmental stages of adolescence? What factors would predict the profiles' generations and possible transition?

In addition, although model parsimony, interpretability, and underlying theoretical logic were taken into consideration to select the final model, it is noteworthy that the entropy of 0.74 in our study did not meet the optimum size of 0.8 (57). Therefore, more studies are required to duplicate the findings of the current study.

Finally, the environmental context should be considered when we interpreted the current findings. In other words, these four distinct solitude groups were identified in the context of urban Chinese culture. As such, the categorizations or the specific performance of solitary groups may vary in different contexts, such as west and east or urban and rural. Accordingly, more diverse research designs, including cross-cultural or cross-region studies, are needed to further explore the implications of solitude.

## 6. Implications

By approaching a person-centered research, the current study sheds light on the cost and benefit of solitude among Chinese late adolescence. The findings have practical implications for individuals, families, and schools. For instance, as we revealed two profiles that may benefit from solitude, adolescents could engage in more activities voluntarily, especially physical activities, when spending time alone. Furthermore, the study focuses on late adolescence, which corresponds to the developmental stage, especially of university freshmen and sophomores. During this period, adolescents often experience a paradoxical phase characterized by a struggle between peer interaction and solitude. As the current findings suggest adolescents in the absence of aloneness group showed the lowest level of psychological maladjustment and the highest level of basic psychological needs satisfaction, it is crucial for schools and parents to properly guide adolescents in understanding and embracing solitude and self-exploration, enhancing their social skills, and thereby safeguarding their mental health.

## Data availability statement

The original contributions presented in the study are included in the article/Supplementary material, further inquiries can be directed to the corresponding author.

## Ethics statement

The studies involving human participants were reviewed and approved by Research Ethics Review Board at Shanghai Normal University. The patients/participants provided their written informed consent to participate in this study.

## Author contributions

TZ, DL, and JL conceptualized the study and developed methods, ethical documentation, and study materials. TZ recruited participants and collected data. TZ and LL drafted the manuscript. TN, DL, and JL conducted the proofreading. JL funded the current project and supervised the whole manuscript writing process. All authors contributed to the article and approved the submitted version.
