# Supplementary material for: Solitude profiles and psychological adjustment in Chinese late adolescence: a person-centered research
Source: Front Psychiatry. 2023 Jul 6;14:1173441. doi: 10.3389/fpsyt.2023.1173441 (PMC10358355; doi:10.3389/fpsyt.2023.1173441)
Supplement: Supplementary file 1 [file Table_1.DOCX]

**Appendix**

**Table.** Item standardized loadings for adolescents’ solitary behavior measure from the exploratory factor analysis (pilot sample, *N* =228)

| **Items** | **Self-reflection** | **Problem-solving** | **Physical Activities** | **Leisure browsing** |
| --- | --- | --- | --- | --- |
| When I am alone, I would like… |  |  |  |  |
| 1. to think about my future. | **0.84*** | 0.02 | 0.01 | 0.02 |
| 2. to think about who I am as a person. | **0.85*** | -0.11* | -0.03 | -0.04 |
| 3. to think about what I am going to do. | **0.80*** | -0.01 | 0.03 | 0.16* |
| 4. to think about what kind of life I want to lead in the future | **0.76*** | -0.02 | 0.04 | 0.04 |
| 5. to reflect on my strengths and weaknesses | **0.68*** | 0.08 | 0.09 | -0.02 |
| 6. to think about what I like and what I don't like. | **0.75*** | 0.02 | -0.07 | 0.01 |
| 7. to think about how the things around me affect my state. | **0.69*** | 0.05 | -0.02 | -0.05 |
| 8. to complete my homework. | -0.06 | **0.91*** | -0.05 | -0.03 |
| 9. to work on unfinished tasks. | 0.00 | **0.87*** | -0.01 | 0.01 |
| 10. to solve problems at hand. | 0.05 | **0.73*** | 0.05 | 0.05 |
| 11. to learn professional knowledge and skills. | 0.08 | **0.60*** | 0.02 | 0.06 |
| 12. to go to the gym. | 0.07 | -0.06 | **0.71*** | 0.04 |
| 13. to use fitness software or videos to exercise. | -0.08 | 0.02 | **0.72*** | 0.03 |
| 14. to go to play ball/do yoga. | -0.04 | -0.02 | **0.59*** | -0.08 |
| 15. to go running. | 0.18* | 0.15* | **0.45*** | -0.09 |
| 16. to browse articles or information. | 0.19* | 0.04 | -0.05 | **0.69*** |
| 17. to watch videos. | -0.03 | -0.05 | -0.02 | **0.63*** |
| 18. to read non-professional books (e.g., novels, magazines, etc.). | -0.02 | 0.22* | 0.02 | 0.29* |
| 19. to browse social networks. | -0.02 | 0.02 | 0.04 | **0.83*** |

*Note*. Loadings larger than .30 are in bold. * *p* < .05.
